# Highly efficient adsorptive removal of the carcinogen aflatoxin B_1_ using the parasitic plant *Cuscuta corymbosa* Ruiz & Pavon

**DOI:** 10.1007/s11356-023-30992-w

**Published:** 2023-11-24

**Authors:** Alma Vázquez-Durán, María de Jesús Nava-Ramírez, Rubén Martínez-Escutia, Juan de Dios Figueroa-Cárdenas, Carlos López-Coello, Guillermo Téllez-Isaías, Abraham Méndez-Albores

**Affiliations:** 1 Unidad de Investigación Multidisciplinaria (UIM) L14 (Alimentos, Micotoxinas, y Micotoxicosis), Facultad de Estudios Superiores Cuautitlán (FES-C), Universidad Nacional Autónoma de México (UNAM), Cuautitlán Izcalli, State of Mexico 54714 Mexico; 2 CINVESTAV-Unidad Querétaro, Libramiento Norponiente No. 2000, Fraccionamiento Real de Juriquilla, 76230 Querétaro, Mexico; 3 Departamento de Medicina y Zootecnia de Aves, Facultad de Medicina Veterinaria y Zootecnia, Universidad Nacional Autónoma de México, 04510 Mexico City, Mexico; 4 Division of Agriculture, Department of Poultry Science, University of Arkansas, Fayetteville, AR 72701 USA

**Keywords:** Aflatoxin B_1_, Plant-based adsorbent, Parasitic plant, Separation technology

## Abstract

The ever-growing consumption of herbs around the globe has motivated the researchers to acquire practical knowledge about other potential applications in human and animal health. In this research, an unmodified adsorbent prepared from the holoparasitic herb *C. corymbosa* was utilized for the removal of the carcinogen aflatoxin B_1_ (AFB_1_) from aqueous solutions. The adsorbent was characterized by Fourier transform near-infrared/mid-infrared spectrophotometry (FT-NIR/MIR), environmental scanning electron microscopy (ESEM), energy-dispersive X-ray fluorescence spectroscopy (EDX), X-ray diffraction (XRD), and point of zero charge (pH_pzc_). Adsorption experiments were carried out in batch systems, and the experimental data was used for isothermal (Langmuir and Freundlich) and kinetic (linear and non-linear forms of the pseudo-first and pseudo-second order) models. In general, the unmodified adsorbent removed AFB_1_ independent of the solution pH, showing a theoretical adsorption capacity of 555.76 mg AFB_1_/g at 303 K, significantly higher than that reported for other plant-based adsorbents and comparable with the efficiency of various inorganic adsorbents. Non-electrostatic attractions such as hydrogen bonding and dispersion forces along with complexation mechanisms were the primary interactions responsible for the adsorption of the pollutant. Our results clearly show that *C. corymbosa* could be a promising material for practical adsorption applications in the drinking water industry.

## Introduction

Mycotoxins are fungal secondary metabolites that represent a rising hazard for public and animal health. Up to now, nearly 400 toxic compounds produced by more than a hundred fungal strains have been discovered (Jard et al. 2011). Mycotoxins of most concern are mainly produced by fungi belonging to three genera: *Aspergillus*, *Fusarium*, and *Penicillium* (Reddy et al. 2010). Among the mycotoxins synthesized by these fungi, aflatoxins, deoxynivalenol, fumonisins, zearalenone, ochratoxin, and T-2 toxin are considered the most poisonous compounds to mammals with significant economic importance in agriculture. Aflatoxins have been extensively studied for their adverse effects on humans and animals. The most significant toxin in this important group of mycotoxins is aflatoxin B_1_ (AFB_1_), one of the most potent naturally occurring human carcinogens (Ostry et al. 2017).

Aflatoxins have been widely studied because they contaminate food and feedstuffs, but little is known about their presence and fate in water sources. It has been reported that aflatoxins can be transported by rainfall to ground water and subsequently to surface water (Manjengwa et al. 2019). Aflatoxins have also been detected in bottled water; however, the concentration only ranged from 0.22 to 0.70 ng/L (Mata et al. 2015). The contamination levels reported may be considered negligible; nevertheless, the long-term consumption of aflatoxin-polluted drinking water may pose a significant risk to human health (Picardo et al. 2019). Although the maximum levels of aflatoxins in certain commodities are well-established in many developed countries, specifications have not yet been made for drinking water. Regulatory authorities and food industries implement strict monitoring and control measures to mitigate the risks associated with aflatoxin contamination. To prevent the entry of aflatoxins into the food/feed/water chain, many strategies have been proposed. These strategies aim to reduce or eliminate aflatoxin levels in the contaminated products. While complete elimination is challenging, several strategies based on physical, chemical, and biological approaches have been proven (Ismail et al. 2018; Sipos et al. 2021). Regarding physical strategies, adsorption is a widely used technology to reduce aflatoxin levels in food/feed and water, as it involves the binding of toxins to specific adsorbent materials. Recently, plant-based adsorbents have gained significant attention as natural and sustainable materials for adsorbing aflatoxins (Vázquez-Durán et al. 2022). These adsorbents have shown varying degrees of aflatoxin adsorption efficiency depending on their chemical composition and surface properties. Some studies have reported high aflatoxin binding capacities (Greco et al. 2019), while others have found moderate to low adsorption efficiencies (Fernandes et al. 2019). In general, plant-based adsorbents provide a promising avenue for aflatoxin adsorption due to their renewable nature and potential effectiveness. However, further research is needed regarding the conversion of other agricultural wastes into value-added products since the potential application of plant-based adsorbents for the drinking water industry is still in its infancy.

*Cuscuta corymbosa* Ruiz & Pavon, commonly known as field dodder, is a parasitic plant species belonging to the family Convolvulaceae. Field dodder is native to North and Central America and can be found in various habitats around the globe. In Mexico, *C. corymbosa* has been reported from the state of Nayarit in the north to the state of Oaxaca in the south (McVaugh 1987). As a parasitic plant, *C. corymbosa* attaches itself to the host plants using specialized structures called haustoria, which penetrate the host’s vascular tissue to extract water and nutrients (Shimizu and Aoki 2019). This parasitic relationship can weaken and ultimately damage the host plant, affecting its growth and productivity. Since *C. corymbosa* spread rapidly and intertwine with multiple hosts, its control is difficult. Therefore, prevention and early detection are essential to manage infections; however, physical removal of the parasitic plant must be done once it has established extensive connections. Understanding the biology, ecology, and specially control measures of *C. corymbosa* is crucial for mitigating its impact on crop production and maintaining the health of the affected ecosystems (Dawson et al. 1994). As the main novelty, this research considers the utilization of the genus *Cuscuta* (an aggressive parasitic plant that threatens agriculture worldwide) for the preparation of an unmodified and low-cost adsorbent, combining simplicity and efficiency for the removal of AFB_1_ from water. Another novelty of this work is that AFB_1_ molecules were almost completely removed from the aqueous solution containing the plant-based adsorbent at a low dose just after few minutes of contact time at neutral pH. These characteristics make the adsorbent a technically promising material to be used for the removal of aflatoxins in the drinking water industry. To the best of our knowledge, there are no studies on the use of the holoparasitic genus *Cuscuta* as an adsorbent for mycotoxins. Consequently, the objective of this study was to (1) prepare and characterize an unmodified adsorbent from *C. corymbosa* and (2) examine the possibility of using the as-prepared adsorbent for the removal of AFB_1_ in aqueous media.

## Experimental section

### Adsorbate

AFB_1_ standard from *Aspergillus flavus* with a molecular weight of 312.27 g/mol was obtained from Merck KGaA, Darmstadt, Germany. The AFB_1_ powder was dissolved in dimethyl sulfoxide (DMSO) to enhance solubility and ensure a homogeneous solution. Subsequently, the AFB_1_ solution was diluted with distilled water to achieve the desired concentration for the adsorption experiments.

### Adsorbent preparation

Fresh *C. corymbosa* samples at the complete growth-stage (flowers and seeds) were collected from infected pepper trees (*Schinus molle* L.) near the Superior Studies Faculty at Cuautitlan (FESC-UNAM, State of Mexico, Mexico). A voucher of the specimen was authenticated by Dr. Alejandro Torres-Montúfar, responsible for the management of the Herbarium of the Biological Sciences Department (NYBG Steere Herbarium code: FESC). The accession number for the specimen was 11971. In the preparation of *C. corymbosa* for its use as an adsorbent material, washed and drained samples were spread in a stainless-steel tray and dehydrated in a conventional solar dryer system following the recommendations of Vázquez-Durán et al. (2021). When the average moisture content reached a value of 7%, the samples were ground with an electric plate-style mill type C-11–1 (Glen Mills Inc., Clifton, NJ, USA), and the finely ground material was screened through a 60-mesh US sieve (particle size of < 250 µm). To maintain the integrity of the adsorbent, samples were stored in vacuum-sealed plastic containers and kept at a temperature of − 20 °C until further analysis.

### Adsorbent characterization

The functional groups on the surface of the adsorbent were identified by Fourier transform near-infrared (NIR)/mid-infrared (MIR) spectrophotometry. A Perkin Elmer Frontier NIR/MIR SP8000 spectrophotometer equipped with an ATR accessory (DuraSamplIR II, Smiths Detection, Warrington, UK) was employed for the analysis. The spectral ranges of 4000 to 400 cm^−1^ and 10,000 to 4000 cm^−1^ were chosen to cover the MIR and NIR regions, respectively. To ensure robust outcomes, 32 scans were performed resulting in improved spectrum quality and a favorable signal-to-noise ratio. A resolution of 4 cm^−1^ was selected in both modes to capture the relevant FT-NIR/MIR absorption bands. The morphological features and the multi-elemental analysis of the adsorbent were investigated using an environmental scanning electron microscope accessorized with energy-dispersive X-ray fluorescence spectroscopy (Phillips XL30/40 EDS-ESEM, Eindhoven, The Netherlands). Microscopy analysis was performed at 750, 2500, and 5000 × with an accelerating voltage of 5 kV. The multi-elemental analysis was evaluated using a high-performance XTrace microspot X-ray source, and the generated X-ray fluorescence spectrum was measured with the attached XFlash® 6/10 silicon drift detector (Bruker Nano GmbH, Berlin, Germany). The X-ray measurements were recorded with a 2100-Rigaku diffractometer (Rigaku Co., Tokyo, Japan). CuKα radiation and a fixed power source of 30 kV and 20 mA were used. The X-ray diffraction patterns were obtained in the region of 2θ from 5° to 70°, using a 0.02° step size. The point of zero charge (pH_pzc_) was determined following the recommendations of Nava-Ramírez et al. (2021). The pH was adjusted to different values by the addition of 0.1 M HCl or NaOH solutions. Finally, photosynthetic pigments (chlorophyll *a*, chlorophyll *b*, and total carotenoids) were extracted with 96% ethanol, and their content was determined spectrophotometrically using a Cary 8454 UV–Vis Diode Array System spectrophotometer (Agilent Technologies, Santa Clara, CA, USA). The absorption coefficients reported by Lichtenthaler and Wellburn (1983) were used for pigments estimation.

### Adsorption studies

Adsorption experiments were conducted in batch mode using a small stackable incubated/refrigerated orbital shaker SHKE6000-7 (Thermo Scientific, Marietta, OH, USA) operated at a speed of 200 rpm. To determine the optimal quantity required for achieving the highest adsorption rate, the adsorbent dose was varied (0.625, 1.25, 2.5, 5, 10, 20, and 40 mg) in 10-mL flasks containing 5 mL of the AFB_1_ solution (0.5 μg AFB_1_/mL) which were agitated at 303 K for 40 min. Afterward, the ideal adsorbent dose was selected for all subsequent trials, and the effects of other factors, such as contact time (2.5, 5, 10, and 20 min), solution pH (2, 5, 7, and 9), solution ionic strength (0.025 and 0.25 M NaCl, CaCl_2_, and MgCl_2_), adsorbate concentration (1, 2, 4, 8, 16, 32, 64, 128, and 256 mg AFB_1_/L), and temperature (303 and 313 K) were analyzed. All experiments were carried out in quintuplicate.

After the completion of the adsorption process, the supernatant was centrifuged (7000 × *g* for 7 min) to separate the adsorbent from the liquid phase. To ensure a clean sample, the supernatant was further filtered using a PTFE membrane syringe filter with a 0.22-µm pore size. The AFB_1_ concentration was estimated using ultra high-performance liquid chromatography (UPLC) as described below. The adsorption capacity of AFB_1_ onto the adsorbent and the adsorption rate was determined using the following equations:

1 $${q}_{e}=\frac{{(C}_{0}-{C}_{e}) V}{m}$$

2 $$\mathrm{Adsorption\;rate}=\frac{{(C}_{0}-{C}_{e})}{{C}_{0}}\times 100$$

In the mathematical expressions, ${C}_{0}$ represents the initial AFB_1_ concentration (µg/L), while ${C}_{e}$ represents the equilibrium concentration of AFB_1_ (µg/L), $V$ symbolizes the volume of the solution (L), and $m$ indicates the mass of the adsorbent (g).

### AFB_1_ determination

The AFB_1_ concentration was determined by ultra-high–performance liquid chromatography (UPLC) using a Waters ACQUITY H-Class System accessorized with an optimized fluorescence detector (Waters, Milford, MA, USA). A chromatographic column (ACQUITY UPLC BEH C18, 2.1 × 100 mm, 1.7 μm) was employed in the experiment to separate the analyte, with an injection volume of 10 μL. The composition of the mobile phase was HPLC grade water/methanol/acetonitrile (64:18:18), and the flow rate was 0.4 mL/min. The excitation and emission wavelengths were 365 and 429 nm, respectively. The system was computer controlled, and the EMPOWER3 software was used for the analysis of the data.

### Method validation

To verify the performance of the UPLC methodology, the guidelines for single-laboratory validation of analytical methods for trace-level concentrations of organic chemicals elaborated by the AOAC/FAO/IAEA/IUPAC (Alder et al. 2000) were used. Four parameters were evaluated: (*i*) limit of detection (LOD), (*ii*) limit of quantification (LOQ), (*iii*) recovery, and (*iv*) linearity. The LOD and LOQ were calculated based on the signal-to-noise ratio. The recovery was determined by evaluating the percent recoveries of AFB_1_ using different contents (from 8 to 250 ng AFB_1_/g) that were added to blank matrix samples. The linearity was determined using the AFB_1_ standard solution over the range of 10 to 1000 ng AFB_1_/L. In general, LOD and LOQ were found to be 2.0 and 6.7 ng AFB_1_/L, respectively. The average recoveries ranged between 92 and 95%, and the linearity estimated with the coefficient of determination within the selected AFB_1_ range was 0.9984. These results indicated that the UPLC methodology was applicable to the determination of AFB_1_.

### Kinetic studies

The kinetic study was conducted using the ideal adsorbent dose and pH. The temperature was fixed at 303 K, and the AFB_1_ concentration was 0.5 μg/mL. The linear and non-linear forms of the pseudo-first-order and pseudo-second-order models were used for fitting the experimental data using the following equations:

Linear:

3 $$\mathrm{ln }\left({q}_{e}-{q}_{t}\right)=\mathrm{ln}\;{q}_{e}-{k}_{1}t$$

4 $$\frac{1}{{q}_{t}}= \frac{1}{{k}_{2}{q}_{e}^{2}}+\frac{1}{{q}_{e}}$$

Non-linear:

5 $${q}_{t}={q}_{e} (1-{e}^{{-K}_{1}t})$$

6 $${q}_{t}=\frac{{k}_{2}{{q}_{e}^{2}t}}{1+{k}_{2}{q}_{e}t}$$

In the models, ${q}_{e}$ and ${q}_{t}$ are the capacities of the sorbent to AFB_1_ uptake (mg/g) at the equilibrium and at time $t$ (min), respectively. ${k}_{1}$ and ${k}_{2}$ are the pseudo-first-order and pseudo-second-order apparent adsorption rate constants (1/min and g/mg min), respectively.

### Isothermal studies

For the equilibrium study, the AFB_1_ concentration ranged from 0.5 to 256 mg/L, and the experimental runs were performed at 303 and 313 K. The non-linear methods of Langmuir and Freundlich isotherms were used to estimate the maximum adsorption capability of the adsorbent under optimized experimental conditions, according to the following equations:

7 $${q}_{e}=\frac{{q}_{max}{{K}_{L}C}_{e}}{(1+{K}_{L} {C}_{e})}$$

8 $${q}_{e}={K}_{F}{C}_{e}^{{~}^{1}\!\left/ \!{~}_{n}\right.}$$

In the mathematical expressions, ${q}_{e}$ is the amount of AFB_1_ adsorbed per unit weight of adsorbent at equilibrium (mg/g), ${q}_{max}$ is the maximum AFB_1_ capacity (mg/g), ${K}_{L}$ is the Langmuir isotherm constant and is related to the adsorption energy, ${C}_{e}$ is the AFB_1_ concentration in the supernatant at equilibrium (μg/mL), and ${K}_{F}$ (L/mg) and $n$ are the Freundlich isotherm constants related to the sorption capacity and intensity, respectively.

### Experimental design and statistical analysis

The statistical analysis was performed using the Minitab 16.0.1 software (Penn State University, State College, PA, USA). Data were firstly examined with the Ryan-Joiner and Bartlett tests to check the assumptions related to normality and homoscedasticity, respectively. Subsequently, one-way ANOVA was used in a completely randomized design. The mean comparison was performed using the Tukey honest significant difference post hoc test, and a value of *p* < 0.05 was considered to reject the null hypothesis.

## Results and discussion

### Characterization of the adsorbent

#### FT-MIR/NIR spectroscopy

**Fig. 1:**
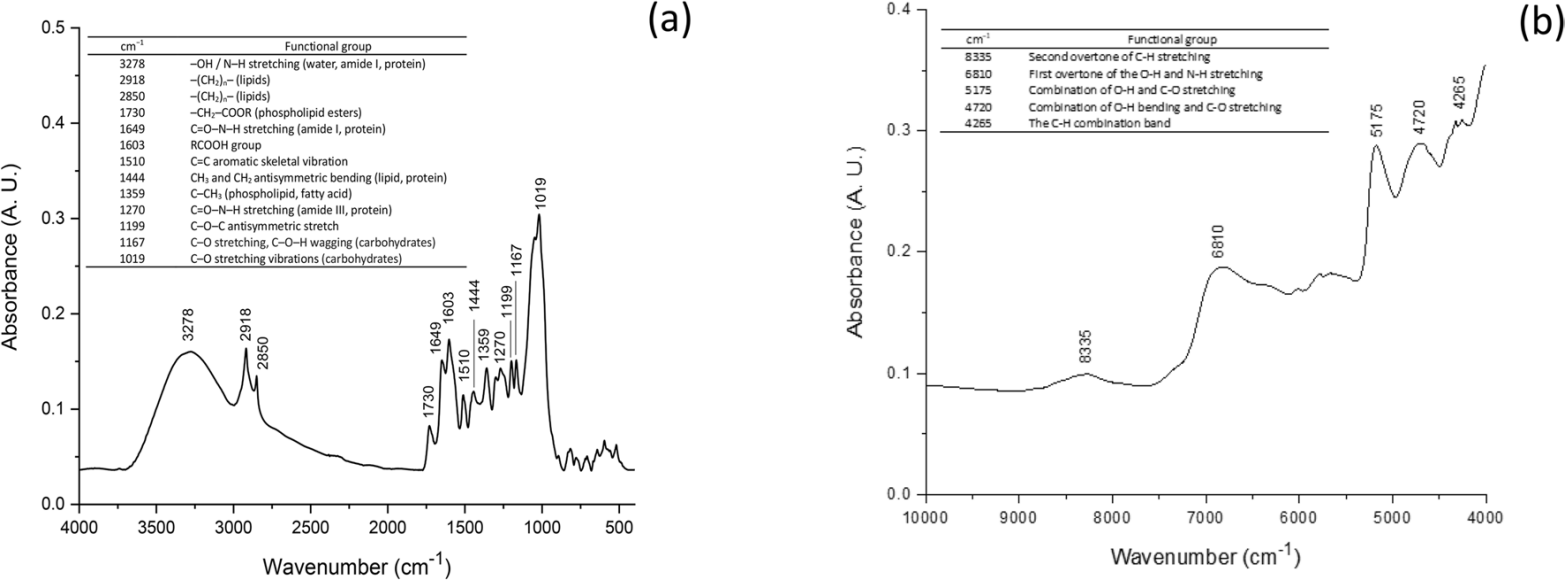
FT-MIR **a** and FT-NIR **b** spectra of the *C. corymbosa* adsorbent

In the present study, FT-MIR/NIR spectroscopy was used to explore the presence of various functional groups on the surface of the adsorbent. In the FT-MIR spectrum (Fig. 1a), the extending vibration of the O–H band occurred at 3278 cm^−1^, alkyl chains (C–H stretching) appeared at 2918 and 2850 cm^−1^, and the carboxyl group at a wavenumber of 1603 cm^−1^. Moreover, the absorption band at 1510 cm^−1^ represents the aromatic ring framework vibration (C = C stretching), and the strong molecular vibration of the C–O bond in carbohydrates appeared at approximately 1019 cm^−1^. On the other hand, Fig. 1b, shows the FT-NIR spectrum of the adsorbent from the 10,000–4000 cm^−1^ wavenumber range. Several important bands were also observed in the spectrum, for instance: the second overtone of the C–H stretching, the first overtone of the O–H and N–H stretching, and the first overtone of the C–H stretching vibrations which were observed at 8335, 6810, and 5127 cm^−1^, respectively. Moreover, two additional absorption bands were observed in the combination region. The O–H bond combined with the C–O bond was detected at 4720 cm^−1^, and the C–H bond combined with the C–H bond appeared at 4265 cm^−1^ (Hong et al. 2019; Chang et al. 2020). A summary of these and other main FT-MIR/NIR bands and their corresponding assignations is depicted in the table located in the up-left inset of Fig. 1. In general, the adsorbent exhibited higher quantities of chemical functional groups, which could participate in the adsorption of the pollutant via electrostatic interactions (Ramales-Valderrama et al. 2016; Zavala-Franco et al. 2018; Nava-Ramírez et al. 2021).

#### ESEM and EDX

**Fig. 2:**
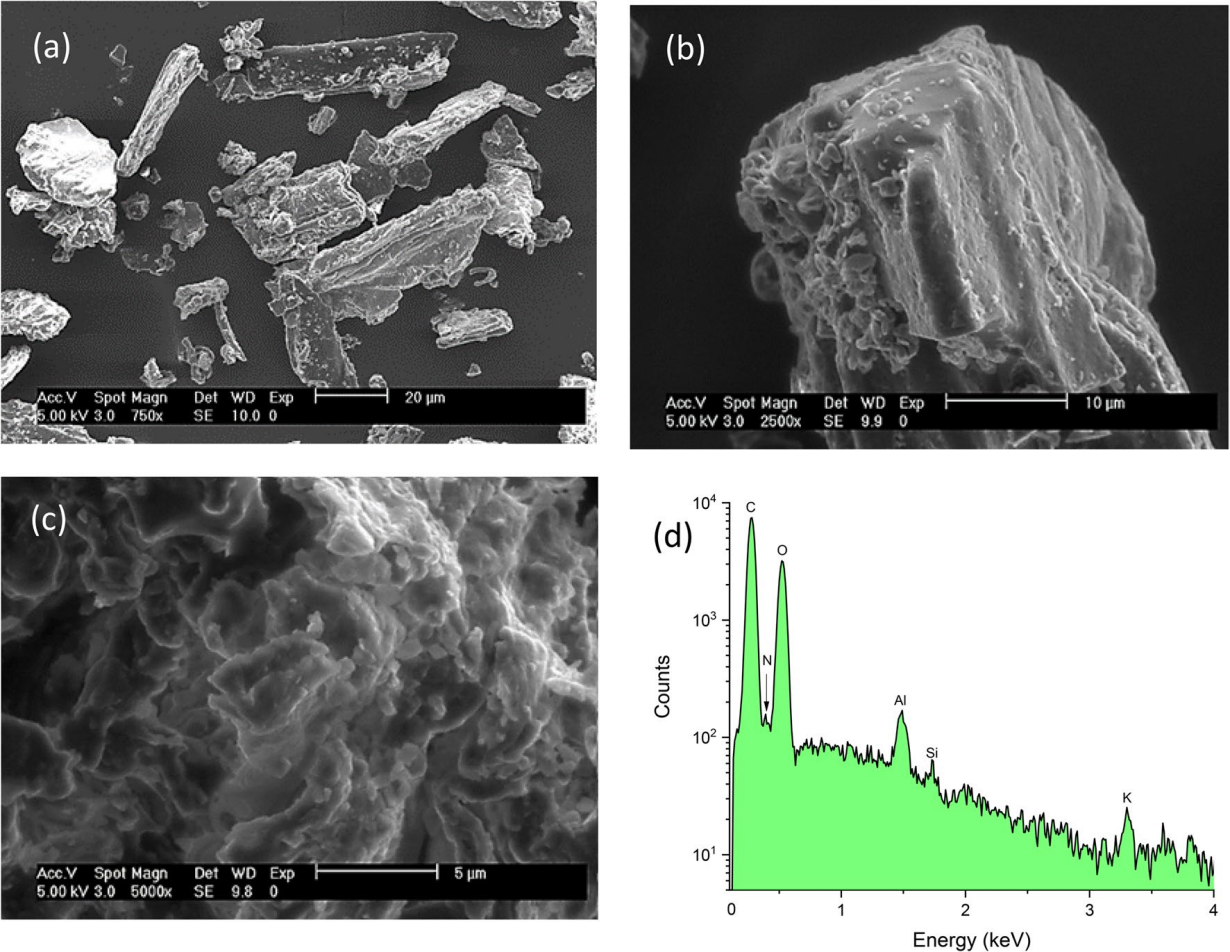
ESEM micrographs of the *C. corymbosa* adsorbent at 750 × (**a**), 2500 × (**b**), and 5000 × (**c**), and the energy-dispersive X-ray spectroscopy (EDX) spectrum

Electron microscopy has been widely utilized to study the morphological features of adsorbent materials. Analysis of the ESEM micrographs at 750 × and 2500 × magnification (Fig. 2a and b) clearly revealed that the adsorbent has a heterogeneous surface. It was also evident that the adsorbent showed an uneven texture along with a lot of irregular surfaces. However, at 5000 × magnification (Fig. 2c), the micrograph showed irregular surfaces with cavities of several sizes, where there is a good chance for AFB_1_ molecules to be adsorbed. These cavities on the adsorbent surface provide channels for the diffusion of AFB_1_ molecules where they can interact with the surface functional groups. These morphological features are suggestive of a material with good adsorptive capacity. On the other hand, the micro-elemental analysis of the adsorbent was accomplished by the energy-dispersive X-ray fluorescence technique. Figure 2d shows the EDX pattern of the adsorbent material. The micro-elemental analysis showed the existence of significant quantities of carbon (51.24%) and oxygen (43.52%). Other signals of minor intensities were also found to be present in the sample, such as nitrogen (4.62%), aluminum (0.54%), silicon (0.02%), and potassium (0.06%). In general, significant carbon, oxygen, and nitrogen contents were present in the adsorbent material (up to 99.38%) which is essential for the adsorptive removal of the pollutant (Saravanan et al. 2021).

#### X-ray diffraction and point of zero charge

**Fig. 3:**
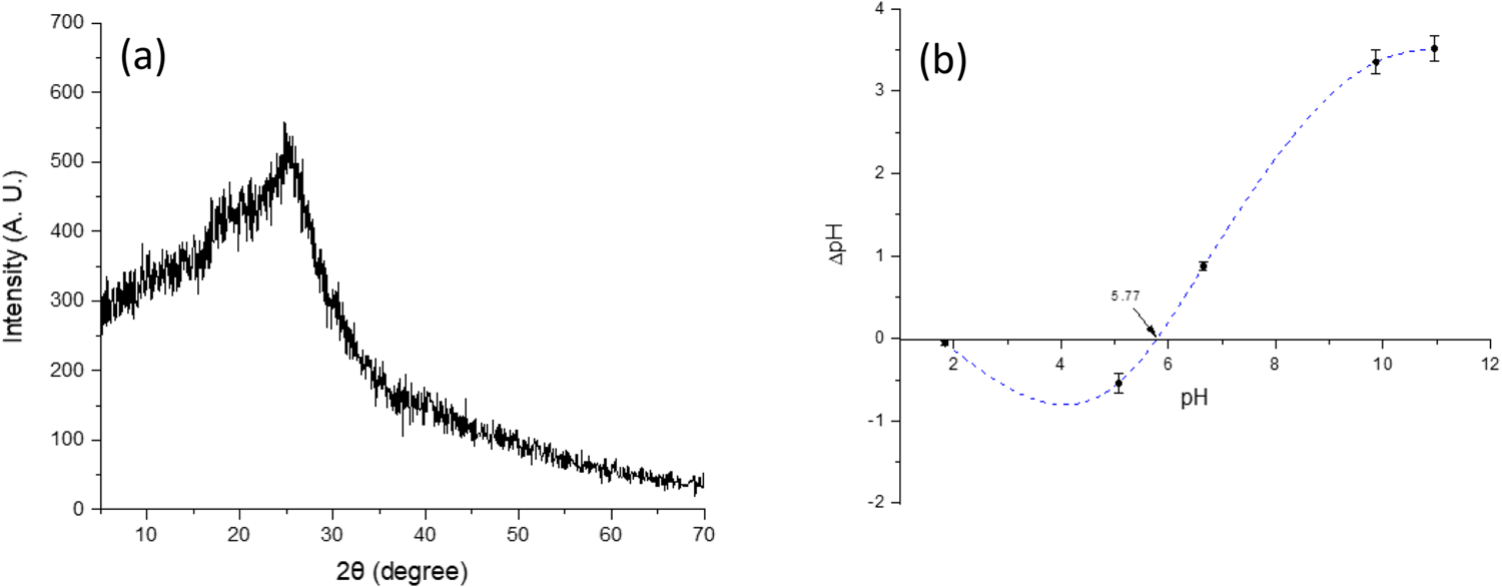
Representative X-ray diffraction pattern (**a**) and point of zero charge (**b**) of the *C. corymbosa* adsorbent

The X-ray diffraction pattern of the adsorbent is shown in Fig. 3a. In general, the diffractogram illustrates the presence of a main diffraction peak at 2θ = 24.7°, which is commonly assigned to the cellulose and hemicellulose crystalline structure (Chen et al. 2012). This result was well corroborated by the FTIR measurements, since the carbohydrate band located at 1019 cm^−1^ was the dominant macromolecular component, which includes cellulose, hemicellulose, starch, sugar, and lignin (Ramales-Valderrama et al. 2016). In this research, the established technique to measure the point of zero charge was the potentiometric titration, and the results are shown in Fig. 3b. As can be seen, the point of zero charge (pH_pzc_) of the adsorbent was found to be 5.77; consequently, the relative surface of the adsorbent is positively charged for pH values below the pH_pzc_ and negatively charged for pH values above 5.77. In other words, the adsorption of anionic molecules via electrostatic interactions is favorable at pH values lower than the pH_pzc_, and the adsorption of cationic molecules is favorable when the solution pH is higher than the pH_pzc_.

#### Photosynthetic pigments

In this research, two main pigment groups: chlorophylls (chlorophyll *a* and chlorophyll *b*) and carotenoids (xantophyll + carotenes) were extracted with 96% ethanol, and their contents were determined spectrophotometrically using the absorption coefficients reported by Lichtenthaler and Wellburn (1983). The results obtained pointed out that the adsorbent prepared from *C. corymbosa* had considerable quantities of chlorophylls. In general, chlorophyll was present as chlorophyll *a* and chlorophyll *b* in the ratio of 1.5:1. The total chlorophyll content was 102 µg/g dry weight. Moreover, significant contents of total carotenoids were present in the adsorbent, reaching values up to 168.37 µg/g dry weight. It has been previously reported that under certain circumstances, the parasitic plant may contain significant amounts of chlorophyll and appear quite green. For instance, van der Kooij et al. (2005), using 80% acetone as an extractant agent, reported a chlorophyll content of 111 µg/g in *C. reflexa* and a carotenoid level of 177 µg/g in *C. campestris*. These results are consistent with our findings.

### Adsorption performance against AFB_1_

#### Adsorbent dose

**Fig. 4:**
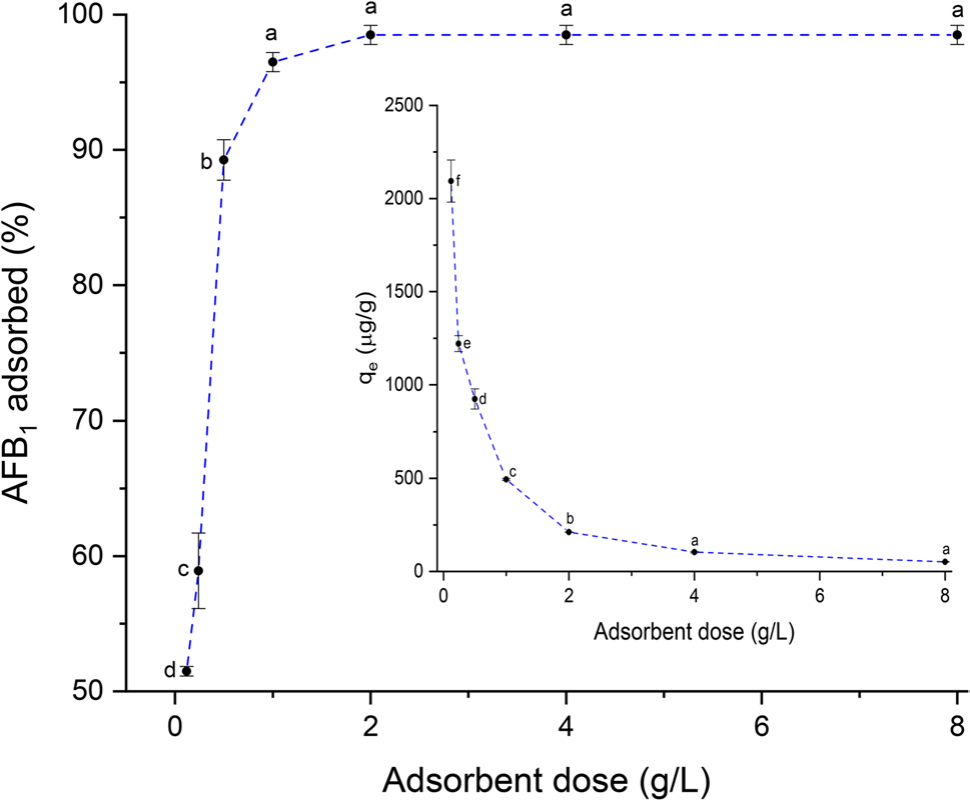
The effect of the adsorbent dose on the removal rate and adsorption capacity of AFB_1_ (contact time 40 min; 0.5 μg AFB_1_/mL; pH 7; and temperature 303 K). For each adsorption parameter, means values not sharing a common superscript letter(s) are significantly different (*p* < 0.05)

One of the parameters that strongly affect the removal rate of the contaminant is the adsorbent dose, as it can determine the percentage of AFB_1_ adsorbed and ultimately can be used to predict the cost of the adsorption treatment. Figure 4 shows the effect of the adsorbent dose on the removal of AFB_1_. In general, the AFB_1_ removal rate increased progressively from 51 to 97% by increasing the adsorbent dose from 0.125 to 1 g/L. However, no significant effect on the removal rate (*p* > 0.05) was attained when using the adsorbent up to 8 g/L (98%), due to spatial inhibition of the adsorptive sites in the presence of an extra dose of the adsorbent material. Consequently, an optimized adsorbent dose of 1 g/L (${q}_{e}$= 484.4 µg/g) was selected for pH optimization.

#### Solution pH

**Fig. 5:**
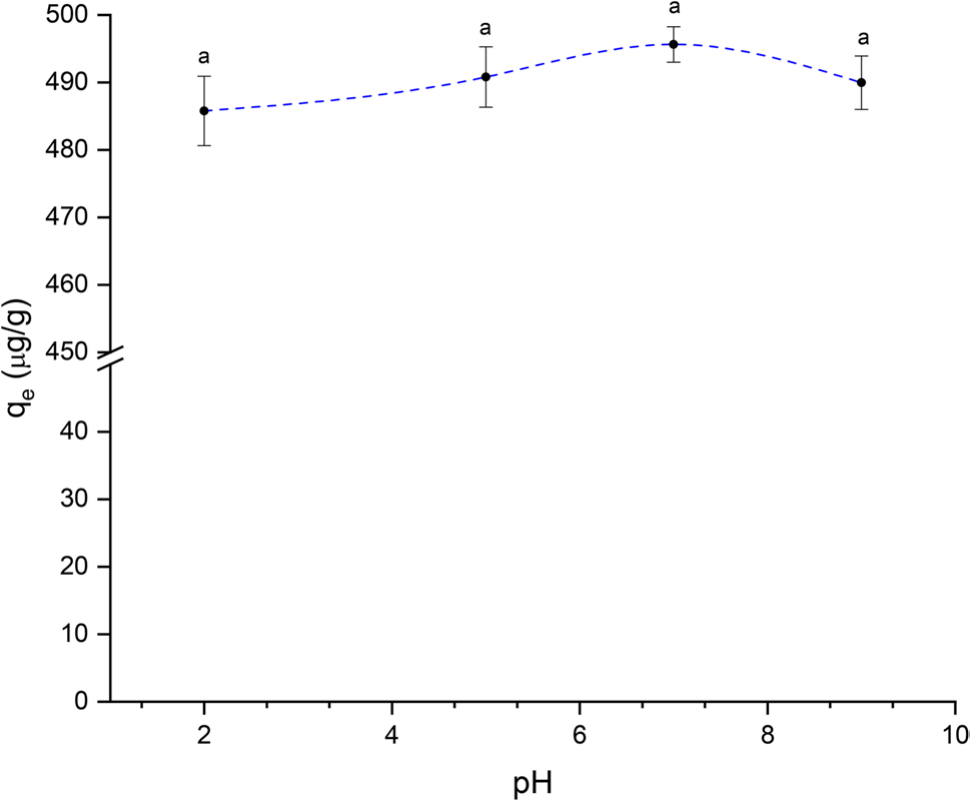
The effect of solution pH on the adsorption capacity of AFB_1_ (adsorbent dose 1 g/L; contact time 40 min; 0.5 μg AFB_1_/mL; and temperature 303 K). Means values not sharing a common superscript letter(s) are significantly different (*p* < 0.05)

Solution pH impacts both the removal rate and the adsorption capacity since pH significantly affects the adsorbate species distribution in water as well as the ionization of the functional groups present on the surface of the adsorbent material. Figure 5 shows the adsorption capacity of AFB_1_ on the studied adsorbent as a function of the solution pH (in the pH range of 3–9). The results suggest that the adsorption capacity was independent of the solution pH (average ${q}_{e}$= 473.7 ± 16.4 µg/g). This phenomenon could be mainly understood by the surface charge on the adsorbent material rather than the charge on the AFB_1_ molecule. The charge of the AFB_1_ molecule depends on its acid dissociation constant (pKa), and the AFB_1_ molecule has a pKa value of 17.78 (Peng et al. 2018). Thus, AFB_1_ is neither protonated nor deprotonated within the pH range of this work. In this context, Avantaggiato et al. (2014) reported that grape pomace efficiently adsorbed AFB_1_ (83%) in a similar pH range. These findings clearly suggest that *C. corymbosa* mainly adsorb AFB_1_ via non-electrostatic interactions (hydrogen bonding, dipole–dipole, and hydrophobic interactions). In this research, since pH did not affect AFB_1_ adsorption, neutral pH was selected for the subsequent experiments.

#### Contact time

**Fig. 6:**
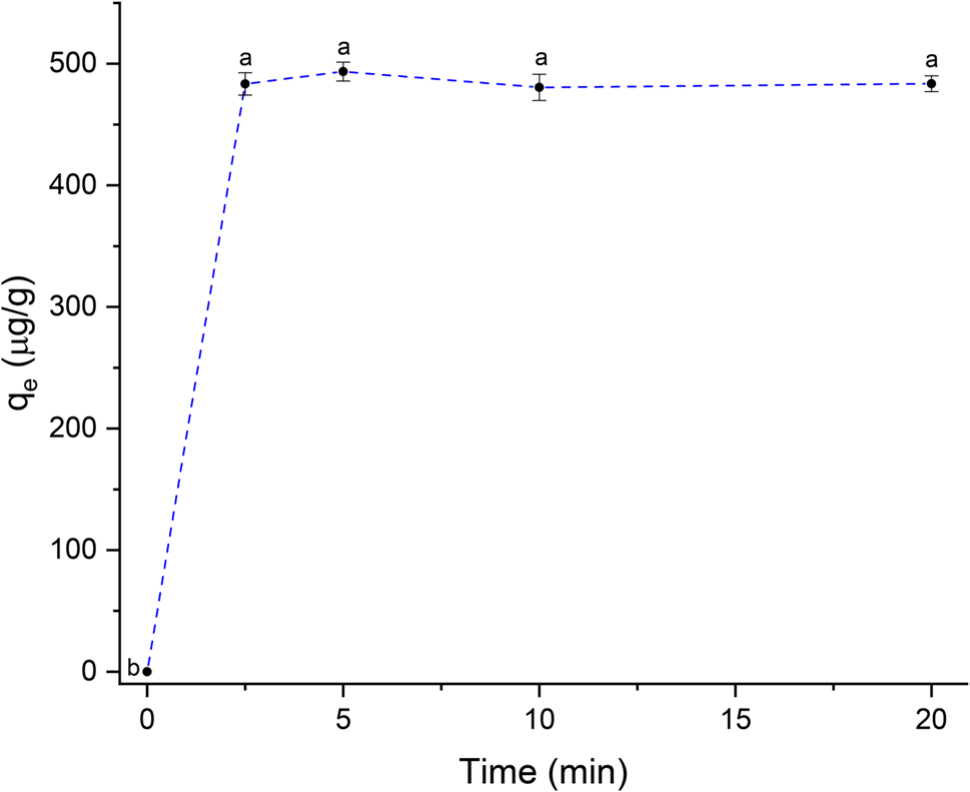
The effect of contact time on the adsorption capacity of AFB_1_ (adsorbent dose 1 g/L; 0.5 μg AFB_1_/mL; pH 7; and temperature 303 K). Means values not sharing a common superscript letter(s) are significantly different (*p* < 0.05)

Adsorption experiments were carried out using an adsorbent dose of 1 g/L, 0.5 μg AFB_1_/mL, pH 7, and a temperature of 303 K. The adsorption capacity was determined after 2.5, 5, 10, and 20 min. Figure 6 shows that the adsorption capacity can be clearly divided in two phases: very rapid and relatively slow. The very rapid adsorption stage occurred from 0 to 2.5 min, in which the adsorption capacity increased rapidly up to 486.3 μg/g. After 2.5 min and up to 20 min, the adsorption capacity reached a plateau (Fig. 6), and no significant changes in the adsorption capacity were observed. Thus, the uptake of the AFB_1_ molecules increased at a very short time (2.5 min) due to the existence of abundant and available adsorptive sites in the adsorbent material. In this regard, Shar et al. (2016) reported that the adsorption of AFB_1_ into banana peel occurred in less than 10 min. To our knowledge, this is the first report showing that *C. corymbosa* can efficiently adsorb AFB_1_ in a very short period, pointing out its potential utility in reducing the availability of AFB_1_ in a real-world scenario.

#### Adsorption kinetics

**Fig. 7:**
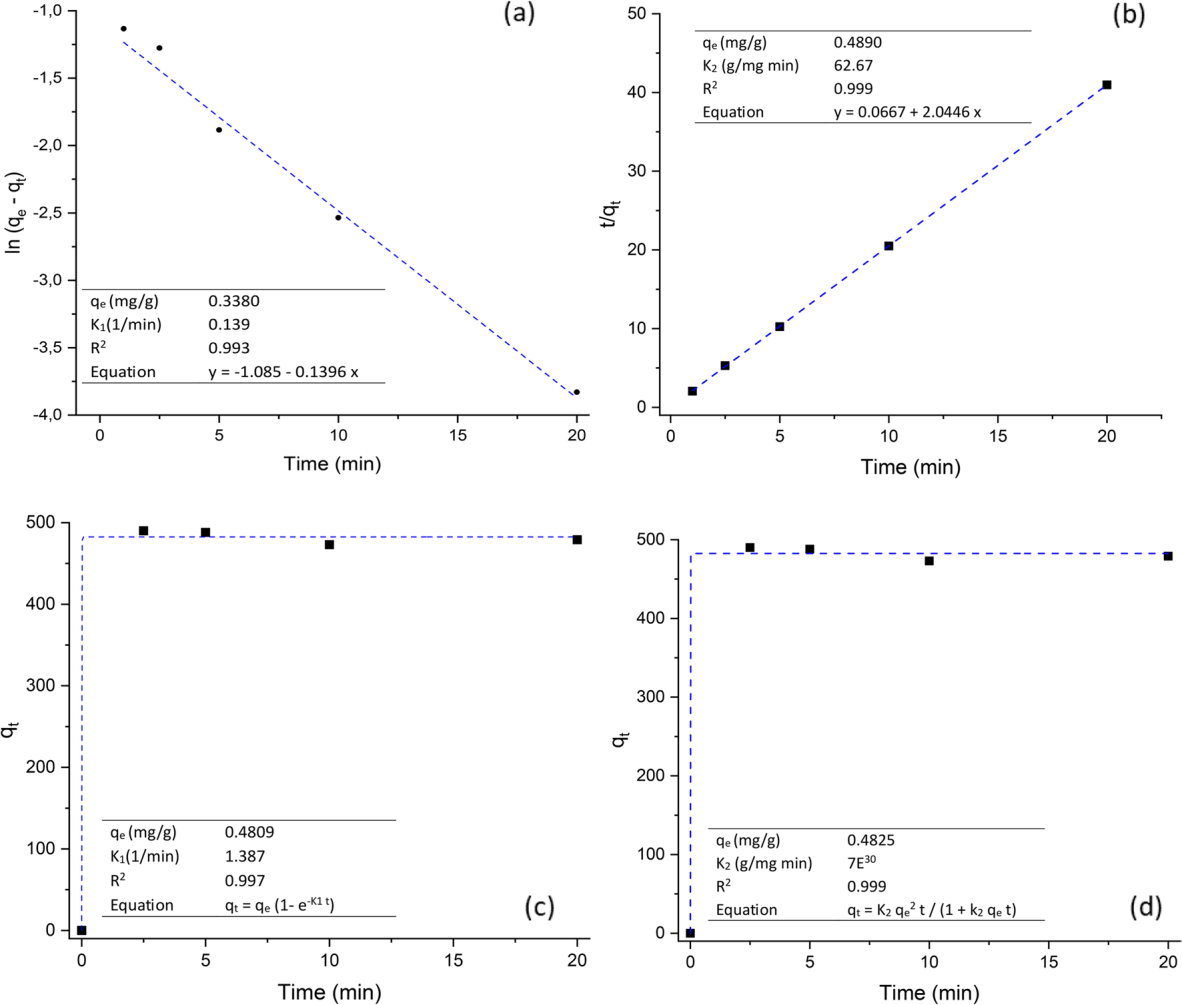
Kinetic models for the adsorption of AFB1 (adsorbent dose 1 g/L; 0.5 μg AFB_1_/mL; pH 7; and temperature 303 K). **a** Linear pseudo-first order, **b** linear pseudo-second order, **c** non-linear pseudo-first order, and (d) non-linear pseudo-second order

Data was examined using the linear and non-linear forms of the pseudo-first-order and pseudo-second-order kinetic models. The calculated parameters are presented in the corresponding table located at the bottom- and the up-left inset of Fig. 7. In general, Fig. 7 and the values of *R*^2^ show that the pseudo-second-order model was better than the pseudo-first-order model in the simulation of kinetics. For instance, the ${q}_{e}$ value (calculated) of the linear pseudo-first-order model (Fig. 7a) was greatly deviated from the experimental value (338.0 vs. 484.4 μg/g). This finding suggests that transformation of non-linear kinetic models to linear forms may change their error distribution, and thus alter their model parameters (Lin and Wang 2009). In this research, the experimental and theoretical ${q}_{e}$ values agreed with each other, as estimated by the non-linear pseudo-second-order kinetic model (Fig. 7d). These results supported the idea that chemisorption was the predominant mechanism during the adsorption process (Ho and McKay 1999). Rasheed et al. (2020) and Li et al. (2020) reported that the kinetics of adsorption of AFB_1_ by blueberry pomace and rice husk follow a pseudo-second-order kinetics model, respectively. Furthermore, Loffredo et al. (2020) reported that coconut fiber, spent coffee grounds, and clementine peel adsorbed the mycotoxin ochratoxin A according to a pseudo-second-order kinetic model. These results are also in accordance with our findings.

#### Solution ionic strength

**Fig. 8:**
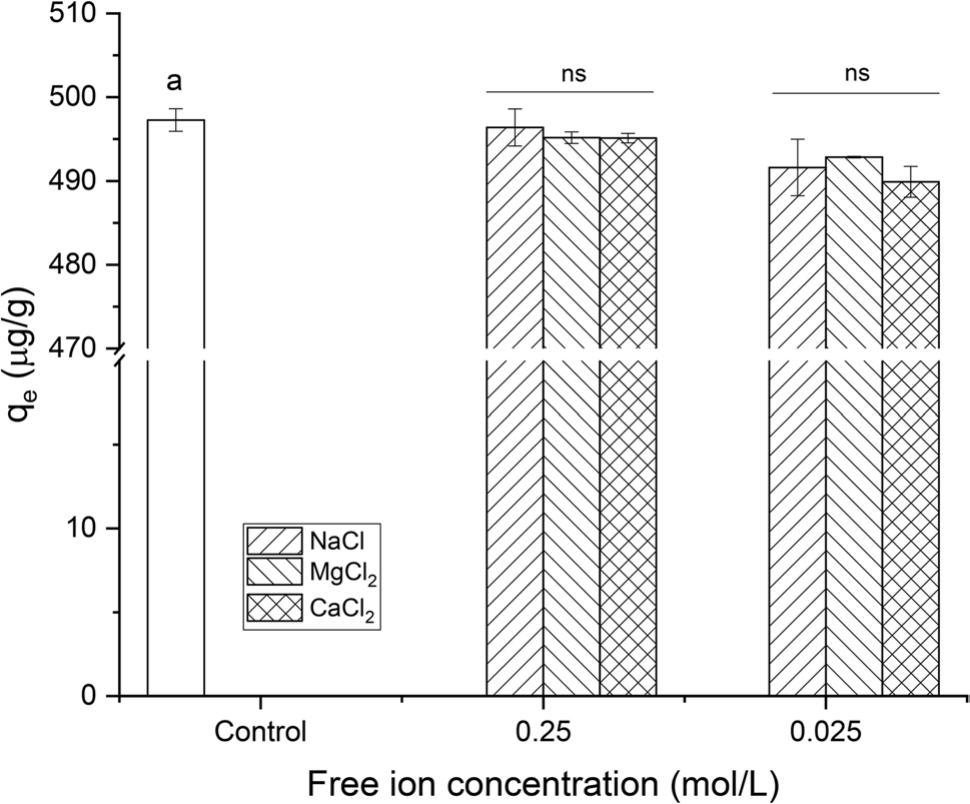
The effect of solution ionic strength on the adsorption capacity of AFB_1_ (adsorbent dose 1 g/L; contact time 2.5 min; 0.5 μg AFB_1_/mL; pH 7; and temperature 303 K). Means values not sharing a common superscript letter(s) are significantly different (*p* < 0.05). ns, not significant

The effect of the ionic strength was also investigated to evaluate the influence of common salts present in water on the AFB_1_ adsorption capacity. For this purpose, the salts evaluated were sodium chloride (NaCl), magnesium chloride (MgCl_2_), and calcium chloride (CaCl_2_) at free ion concentrations of 0.025 and 0.25 mol/L. As shown in Fig. 8, the adsorption capacity was not significantly affected by the presence of the ions since the ${q}_{e}$ values were not statistically different from those found in the control sample. Furthermore, a non-significant effect was also observed as the ion concentration increased from 0.025 to 0.25 mol/L, indicating that ion concentration does not influence the removal of the pollutant. It is well known that solution ionic strength can affect electrostatic interactions due to competition between ions and the pollutant by the available sites of the adsorbent. These experiments allowed us to confirm that electrostatic interactions are not involved due to the insignificant effect of the ionic strength on the AFB_1_ adsorption.

#### Adsorbate concentration

**Fig. 9:**
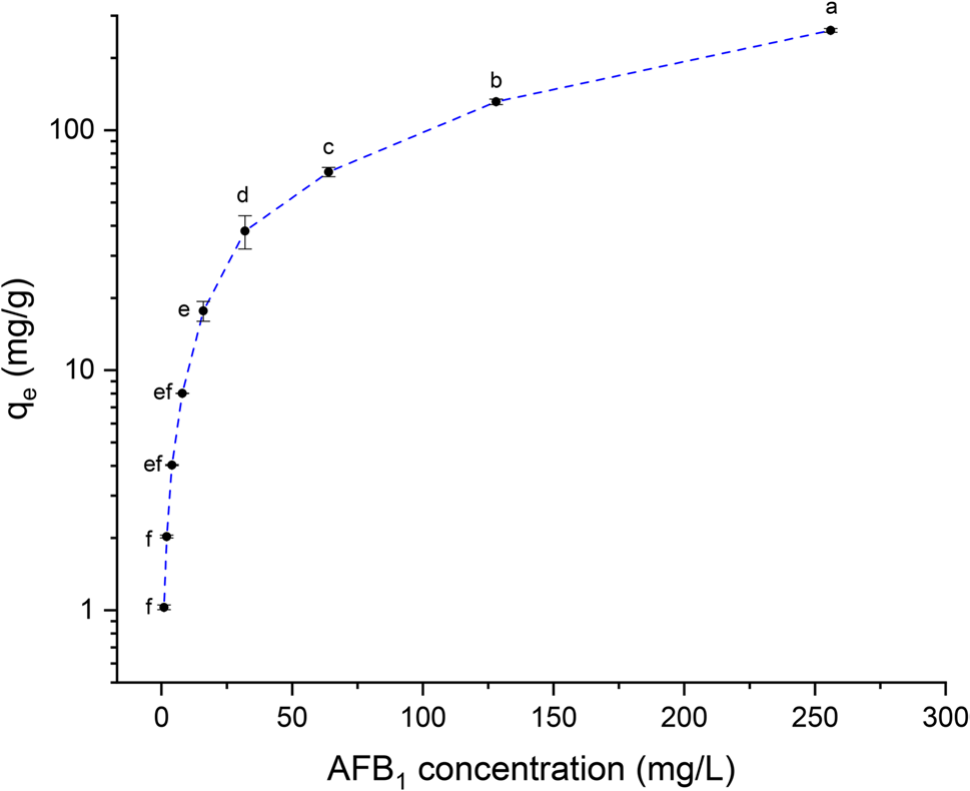
The effect of adsorbate concentration on the adsorption capacity of AFB_1_ (adsorbent dose 1 g/L; contact time 2.5 min; pH 7; and temperature 303 K). Means values not sharing a common superscript letter(s) are significantly different (*p* < 0.05)

Figure 9 shows the effect of different initial concentrations of AFB_1_ (1 to 256 mg AFB_1_/L) on the adsorption capacity. Results clearly indicate that the adsorption process became faster in the initial stages and then progressively decreased; however, the equilibrium was not reached. In general, the adsorption capacity rapidly rose by increasing the AFB_1_ concentration in the solution up to 256 mg AFB_1_/L. Thus, the amount of AFB_1_ adsorbed significantly increased from 1.03 to 254.3 mg/g, suggesting that increasing the initial concentration of the adsorbate also increases the probability of contact between the pollutant and the adsorptive sites (Martínez-Escutia et al. 2022).

#### Isothermal study

**Fig. 10:**
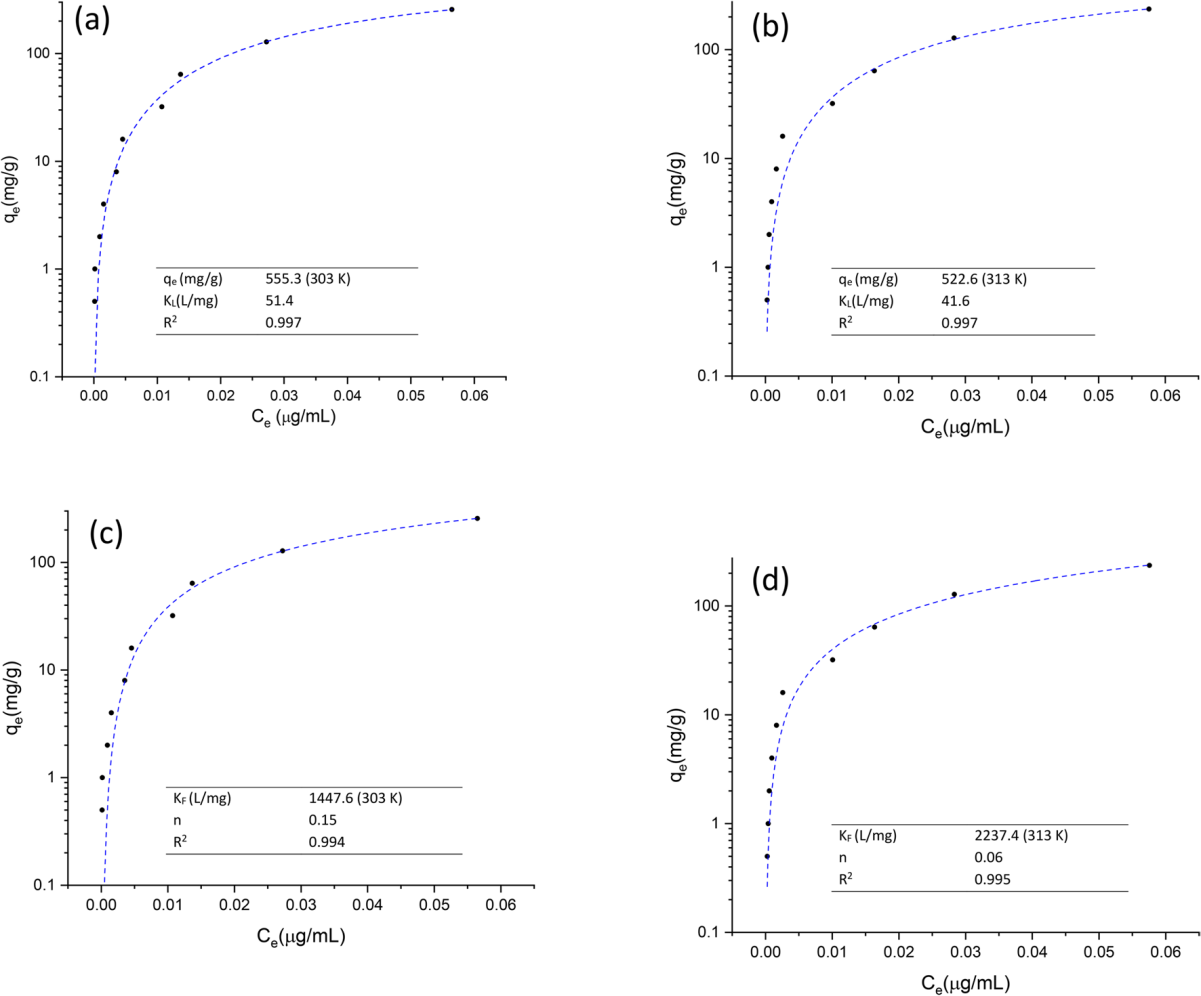
Adsorption isotherms at temperatures of 303 and 313 K: **a**, **b** Langmuir and **c**, **d** Freundlich, respectively

Langmuir and Freundlich’s isotherms were utilized to model the experimental data and calculate the maximum adsorption capacity under optimized conditions at two different temperatures (303 and 313 K). The main isothermal parameters are compiled in the corresponding table located in the bottom-right inset of Fig. 10. In general, the Langmuir isotherm model exhibited a good fit to the experimental equilibrium data, since high coefficient of determination values was observed (*R*^2^ up to 0.997). In general, the Langmuir model assumes that (*i*) one pollutant molecule interacts with only one adsorption site, (*ii*) the adsorption process is accomplished in specific sites, (*iii*) the binding energy is identical at each specific site, and (*iv*) there is no interaction between the pollutant molecules (Cooney 1998). In this research, at a temperature of 303 K, the maximum adsorption capacity attained was 555.76 mg AFB_1_/g, significantly higher than that of various plant-based adsorbents assessed in the recent literature (Table 1). This adsorption capacity of the plant-based binder is comparable with those obtained with various inorganic adsorbents. For instance, Wang et al. (2019) processed a calcium montmorillonite clay with sulfuric acid (18 M) and conducted equilibrium isothermal studies. The authors reported that the acid-processed clay presented a maximum adsorption capacity of 0.37 mol AFB_1_/kg (115.5 mg AFB_1_/g). These results are consistent with our findings. Moreover, in this work, higher *K*_*L*_ values were also found for the Langmuir model, meaning that the pollutant has a strong affinity for the adsorption sites (Hu et al. 2019). To our knowledge, this is the first report in the scientific literature where a plant-based adsorbent is challenged with high concentrations of AFB_1_ in aqueous media. It is pertinent to mention that the studied adsorbent presented a higher adsorption kinetic and accomplished the uptake of AFB_1_ molecules in a very short period. Considering the very low-cost of the adsorbent material, and it is availability worldwide, *C. corymbosa* holds great promise for the removal of AFB_1_ from drinking water.

**Table 1:** Comparison of recent studies for the adsorption of AFB_1_ onto plant-based adsorbents

Adsorbent	Isotherm	Temperature (K)	pH	q_max_ (mg/g)	Reference
Rice husk-based MCM-41	Langmuir	298	NR	0.22	Greco et al. (2019)
Magnetic mesoporous silica from rice husk	Langmuir	283	NR	1.12	Fernandes et al. (2019)
		293	NR	1.05	
		303	NR	0.80	
Citrus fruit peel	Langmuir	310	3	0.08	Avantaggiato et al. (2014)
Blueberry fruit waste	Sips	288	7	4.60	Shar et al. (2016)
Grape pomace	Sips/Hill	310	3	4.73	Rasheed et al. (2020)
			7	4.69	
Banana peel	Langmuir	295	7	0.008	Li et al. (2020)
Olive pomace Grape stems	Langmuir	310	2	3.00	Li et al. (2022)
			7	3.30	
			2	2.50	
			7	2.80	
Sangiovese grape pomace Malvasia grape pomace Almond hull Artichoke	Langmuir	310	3	2.93	Asghar et al. (2022)
			7	2.83	
			3	1.21	
			7	1.43	
			3	2.04	
			7	2.28	
			3	1.79	
			7	1.61	
Durian peel	Langmuir	310	3	3.96	Adunphatcharaphon et al. (2020)
			7	4.06	
Bagasse	Langmuir	NR	NR	66.68	Zahoor and Khan (2018)
Cuscuta	Langmuir	303	7	555.30	This work

*NR* not reported

#### Adsorption mechanism

**Fig. 11:**
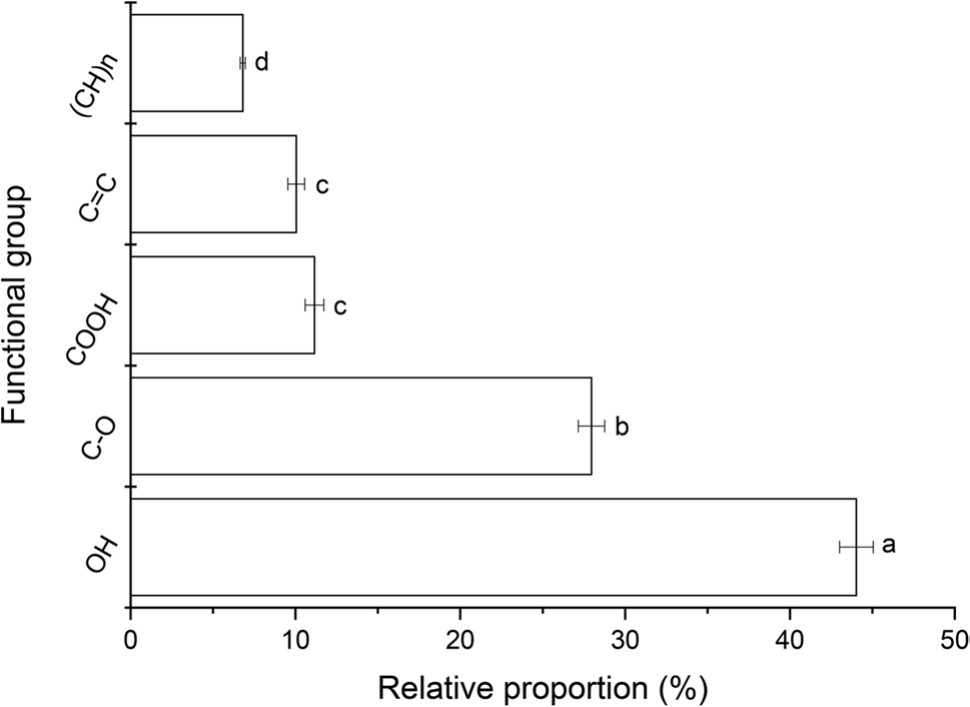
Relative proportion of the principal chemical functional groups of the *C. corymbosa* adsorbent. Means values not sharing a common superscript letter(s) are significantly different (*p* < 0.05)

Based on the recommendations of Vázquez-Durán et al. (2021), the FT-MIR spectrum of the plant-based adsorbent was used to calculate the proportion of the principal chemical functional groups. From Fig. 11, it can be clearly seen that the adsorbent has a significantly higher proportion of OH groups (up to 45%) which can participate in hydrogen bonding with the oxygen atoms of the ether, carbonyl, and methoxy groups in the AFB_1_ molecule. In this work, the adsorption capacity was independent of the solution pH; consequently, hydrogen bonding was considered the dominant force for AFB_1_ adsorption. Moreover, the considerable amount of the C = C groups (around 10%) confirmed the occurrence of π-π stacking between the aromatic rings present on the adsorbent and the AFB_1_ molecule. Thus, dispersion forces are considered the second attraction factor for aromatic rings. Furthermore, our experimental findings also suggest that AFB_1_ is also adsorbed by a complexation mechanism, since it has been previously reported by our research group that photosynthetic pigments (chlorophylls and carotenoids) can form strong non-covalent complexes with the AFB_1_ molecule independent of pH (Nava-Ramírez et al. 2021).

## Conclusion

The present study revealed that the parasitic herb *C. corymbosa* can be a suitable adsorbent for the removal of AFB_1_ from aqueous solutions. The adsorption process was independent of the initial solution pH showing a considerable theoretical adsorption capacity (555.76 mg AFB_1_/g) at 303 K. Moreover, *C. corymbosa* efficiently removed AFB_1_ (up to 97%) using an adsorbent dose of 1 g/L at a very short time (2.5 min). According to the obtained results, the process followed a non-linear pseudo-second-order kinetics, and the Langmuir isotherm model best fitted the experimental equilibrium data. Finally, it is proposed that the combination of non-electrostatic attractions (hydrogen bonding and dispersion forces) along with complexation mechanisms provided strong interactive forces to remove AFB_1_ from water. However, further studies using a continuous column for the removal of aflatoxins in different real drinking water sources need to be conducted. Research in this direction is in progress.

## Data Availability

The datasets used and or analyzed during the current study are available from the corresponding author upon reasonable request.
